# Enhancing performance: unveiling the physiological impact of submaximal and supramaximal tests on mixed martial arts athletes in the −61 kg and −66 kg weight divisions

**DOI:** 10.3389/fphys.2023.1257639

**Published:** 2024-01-12

**Authors:** Aleksandro Ferreira Gonçalves, Bianca Miarka, Clóvis de Albuquerque Maurício, Rafael Pereira Azevedo Teixeira, Ciro José Brito, Diego Ignácio Valenzuela Pérez, Maamer Slimani, Hela Znazen, Nicola Luigi Bragazzi, Victor Machado Reis

**Affiliations:** ^1^ Research Center in Sports Sciences, Health Sciences and Human Development, University of Trás-os-Montes and Alto Douro, Vila Real, Portugal; ^2^ Laboratory of Psychophysiology and Performance in Sports and Combats, Graduate Program in Physical Education, Federal University of Rio de Janeiro, Rio de Janeiro, Brazil; ^3^ Sciences of Physical Activity, Sports and Health School, Faculty of Medical Sciences, Universidad de Santiago de Chile, Santiago, Chile; ^4^ Escuela de Kinesiología, Facultad de Salud, Universidad Santo Tomás, Santiago, Chile; ^5^ School of Public Health, Department of Health Sciences (DISSAL), Genoa University, Genoa, Italy; ^6^ Department of Physical Education and Sport, College of Education, Taif University, Taif, Saudi Arabia; ^7^ Laboratory for Industrial and Applied Mathematics (LIAM), Department of Mathematics and Statistics, York University, Toronto, ON, Canada

**Keywords:** aerobic system, anaerobic system, martial arts, oxidative system, glycolysis

## Abstract

This study delves into the intricate details of Mixed Martial Arts (MMA) by examining key variables such as maximal oxygen uptake (VO2 peak), aerobic energy (EAER), anaerobic energy (EAN), and accumulated O2 deficit (DOA). By investigating associations and comparing athletes in the −61 kg bantamweight and −66 kg featherweight weight divisions, we aim to shed light on their physiological characteristics. The sample consisted of 20 male volunteers separated into two paired groups: ten athletes in the category up to 61 kg (age: 27.7 ± 5.9 years old, height: 170.9 ± 3.4 cm, body mass: 72.8 ± 1.4 kg, fat percentage: 9.5% ± 3.0%, professional experience: 7.5 ± 7.1 years) and ten athletes up to 66 kg (age: 27.6 ± 2.9 years old, height: 176.0 ± 5.5 cm, body mass: 77.0 ± 1.5 kg, fat percentage: 7.85% ± 0.3%, professional experience: 5.5 ± 1.5 years). Remarkably, our findings revealed striking similarities between the two weight divisions. Furthermore, we discovered a negative correlation between VO2 peak and the number of MMA fights, indicating a potential impact of professional experience on aerobic capacity (r = −0.65, *p* < 0.01). Additionally, the number of fights exhibited negative correlations with anaerobic energy (r = −0.53, *p* < 0.05) and total energy cost (r = −0.54, *p* < 0.05). These results provide valuable insights for designing training programs in the context of MMA. While training both weight divisions together can be beneficial, it is equally crucial to incorporate specific weight-class-focused training to address each division’s unique physical demands and requirements.

## Introduction

Mixed Martial Arts (MMA) is a comprehensive combat sport that combines techniques from various martial arts disciplines, encompassing striking (e.g., boxing and kickboxing) (Miarka et al., 2020) and grappling (e.g., Brazilian Jiu-Jitsu and wrestling) (Bello et al., 2019). MMA competitors employ striking and grappling techniques while standing and on the ground (Fernandes et al., 2022). MMA fights are structured into rounds, each lasting several minutes, significantly demanding the athletes’ cardiovascular endurance (Spanias et al., 2019; Gottschall and Hastings, 2023). Adequate cardiovascular endurance is crucial for maintaining a high activity level throughout the fight and facilitating swift recovery between rounds (Ghoul et al., 2019; Kirk and Childs, 2023). Aerobic conditioning is pivotal in enhancing overall performance and endurance among MMA athletes (Kostikiadis et al., 2018; Tota et al., 2019; Pavelka et al., 2020). It enables them to sustain high-intensity efforts over extended durations and aids in recovery between rounds (Kirk and Childs, 2023). However, it is essential to note that the recovery may differ depending on the weight division.

Weight divisions are implemented in MMA to ensure fair competition and mitigate the risk of injuries arising from significant weight disparities (Fares et al., 2019). Each weight division in the UFC has its unique characteristics, fighters, and styles (Ma, 2023). Weight divisions are classified as flyweight (−57 kg), bantamweight (−61 kg), featherweight (−66 kg), lightweight (−70 kg), welterweight (−77 kg), middleweight (−84 kg), light heavyweight (−93 kg), and heavyweight (−120 kg) (UFC, 2023b; UFC, 2023a). The main differences between weight categories typically include the fighters’ size, strength, and fighting style (Fernandes et al., 2022; Folhes et al., 2023). Time-motion analysis can vary significantly between divisions as well (Miarka et al., 2015).

In previous studies, authors grouped the MMA categories into four large groups based on the conditions of technical-tactical similarity (Miarka et al., 2018; Folhes et al., 2023). In the lower weight classes (−61 kg bantamweight and −66 kg featherweight) (−61 kg to −66 kg), athletes tend to be faster and have more strike attempts (Miarka et al., 2018; Folhes et al., 2023), while fighters in the heavier weight classes have more power but fewer strike attempts (Miarka et al., 2018; Folhes et al., 2023). Since there is an increase in the volume of actions for the same amount of time, it is suggested that lighter categories are largely dependent on the aerobic system (Folhes et al., 2023). However, more research on the topic is still needed to clarify in an increasingly precise way what the demands are for the weight categories.

Comparing athletes within weight divisions that are closely matched in terms of weight is essential to maintain a level playing field and preserve the integrity of the sport, particularly during training sessions that involve athletes from different weight divisions (Miarka et al., 2015; Barreto et al., 2019; Dal Bello et al., 2019). Comparing athletes within weight divisions that are closely matched in terms of weight is essential to maintain a level playing field and preserve the integrity of the sport, particularly during training sessions that involve athletes from different weight divisions (Miarka et al., 2015; Barreto et al., 2019; Dal Bello et al., 2019). Many athletes rapidly lose weight in an attempt to gain an advantage over their opponents. This weight reduction can often exceed 10% of the athlete’s initial weight within just 7 days (Crighton et al., 2016; Brandt et al., 2018; Peacock et al., 2022). The methods most commonly employed by athletes aiming to rapidly shed weight include fasting, restricting fluid intake, and utilizing steam rooms and hot tubs (Maurício et al., 2023). These techniques are frequently utilized by combat and sports athletes, despite negative potential effects, such as stomachaches, palpitations, fatigue, cramps, and headaches (Barley et al., 2018; Maurício et al., 2023). Therefore, determining whether there is equivalence in physical capacity can be crucial for transitioning between different weight categories.

When fighters of similar weight and physical conditioning train or compete against each other, it minimizes potential advantages or disadvantages arising from substantial differences in size, strength, and reach (Spanias et al., 2019). Ensuring similar aerobic and anaerobic conditions among athletes allows them to showcase their skills and techniques without encountering excessive physical mismatches (Bray et al., 2021; Chernozub et al., 2022). Furthermore, comparing weight divisions with closely matched weights enhances the safety of the athletes. Considerable weight disparities can lead to severe injuries, as the force generated from strikes or takedowns may be amplified when applied to a smaller opponent (Amtmann, 2003; Miarka et al., 2022). By maintaining closer weight divisions, coaches can reduce the risk of excessive force and potential harm, thereby safeguarding the long-term wellbeing of the fighters (Miarka et al., 2018; Fares et al., 2019; Ignatjeva et al., 2021).

Aerobic conditioning is crucial in improving cardiovascular health, optimizing oxygen utilization, and enhancing the efficient clearance of metabolic byproducts, such as lactic acid (Zebrowska et al., 2019). Various methods are available for assessing aerobic conditioning in MMA athletes. One commonly employed method is submaximal testing (Kruel et al., 2013). These tests estimate athletes’ maximal oxygen uptake (VO2 peak) by analyzing their heart rate response to submaximal exercise (Tota et al., 2019). Conversely, supramaximal or maximum effort tests evaluate an individual’s maximum capacity or performance in a specific activity (Lomax et al., 2019; Andrade et al., 2022). These tests are designed to push individuals beyond their standard capabilities, often exceeding their physical limits (Lomax et al., 2019). In the context of MMA, supramaximal tests can assess an athlete’s physical capabilities and determine their readiness for competition (Andrade et al., 2022). They provide valuable insights into an athlete’s strengths and weaknesses, offering essential information for training and performance enhancement (Ghoul et al., 2019; Kirk et al., 2020). However, it is worth noting that supramaximal and maximal tests typically require specialized laboratory settings, trained personnel, and expensive equipment (Sartor et al., 2013). Moreover, they can be physically demanding and increase the risk of injuries or overtraining (Verellen et al., 2007; Schlegel and Křehký, 2019).

On the other hand, submaximal tests are less demanding and offer a reliable estimation of an athlete’s aerobic fitness without pushing them to their physical limits (Andrade et al., 2022). These tests are more accessible, can be conducted in training facilities or outdoor settings, and require less time (Miller et al., 2019). They provide valuable insights into an athlete’s aerobic capacity and can be used to monitor their progress over time (Andrade et al., 2022).

It is important to emphasize that while aerobic and anaerobic conditioning are critical for MMA athletes, they should be complemented by sport-specific training, strength and power development, flexibility work, and skill acquisition to ensure a well-rounded performance in the sport (Antonietto et al., 2021; Antoniettô et al., 2019; Kirk et al., 2020; Kostikiadis et al., 2018; Spanias et al., 2019; Tota et al., 2019). Therefore, the present research aims to describe variables such as maximal oxygen uptake (VO2 peak), aerobic energy (EAER), anaerobic energy (EAN), and accumulated O2 deficit (DOA) in MMA. Additionally, we aim to establish associations between these variables through submaximal and supramaximal oxygen uptake (VO2 peak) assessments, specifically comparing MMA athletes in the −61 kg and −66 kg weight divisions.

## Methods

### Participants

The present study was conducted in Rio de Janeiro and involved a population of professional athletes in this sport, in accordance with the regulations of the Brazilian Athletic Commission for MMA in 2019. The sample comprised 20 male volunteers who were divided into two paired groups: ten athletes in the up to 61 kg category (with an average age of 27.7 ± 5.9 years, height of 170.9 ± 3.4 cm, body mass of 72.8 ± 1.4 kg, fat percentage of 9.5% ± 3.0%, and professional experience of 7.5 ± 7.1 years), and ten athletes in the up to 66 kg category (with an average age of 27.6 ± 2.9 years, height of 176.0 ± 5.5 cm, body mass of 77.0 ± 1.5 kg, fat percentage of 7.85% ± 0.3%, and professional experience of 5.5 ± 1.5 years).

The process of recruiting the sample occurred during a pre-competitive moment at a UFC high-performance training center in Rio de Janeiro to minimize potential selection bias. All athletes received regular nutritional monitoring and had similar ages, training programs, and diets. None of them were following a weight loss diet. Additionally, the sample size was determined based on previous studies involving combat sports athletes (James et al., 2016; Folhes et al., 2023) and the roster of top-ranked UFC athletes in 2023. This calculation was performed while considering a 95% confidence interval and a 5% margin of error.

Inclusion criteria: Athletes who were not scheduled to fight maintained a balanced diet, and were outside the weight-cutting period; athletes who were outside the specific competitive period of the sport; athletes who had been practicing the sport professionally for at least 1 year; athletes who answered the questionnaire, and; athletes who signed the informed consent form to participate in the study.

Exclusion criteria: Athletes who, for various reasons, could not participate in the physical tests during the data collection period, and; athletes who had pathologies, osteoarticular or musculoskeletal injuries, or other clinical conditions that prevented them from participating in the study.

The procedures followed in this study adhered to the ethical guidelines for research involving human subjects, as outlined in Resolution No. 466/2012 of the National Health Council, which is the governing standard for research involving human participants. These procedures were in line with the principles of the Declaration of Helsinki (2013) of the World Medical Association. All participants were informed about the research procedures and provided their voluntary informed consent by signing the consent form.

### Procedures

During all moments of the present study, participants were instructed to follow their regular diet and sleep hours, avoid drinking caffeine-containing beverages, have a good night’s sleep, and not perform intense physical activities 48 h before the tests.

All subjects performed two stress tests on a horizontal treadmill, one submaximal and one supramaximal. The succession of tests for each subject was always a submaximal test followed by a supramaximal test. The minimum interval between successive tests was 48 h, and the maximum interval of 96 h.

### Submaximal test

**FIGURE 1:**
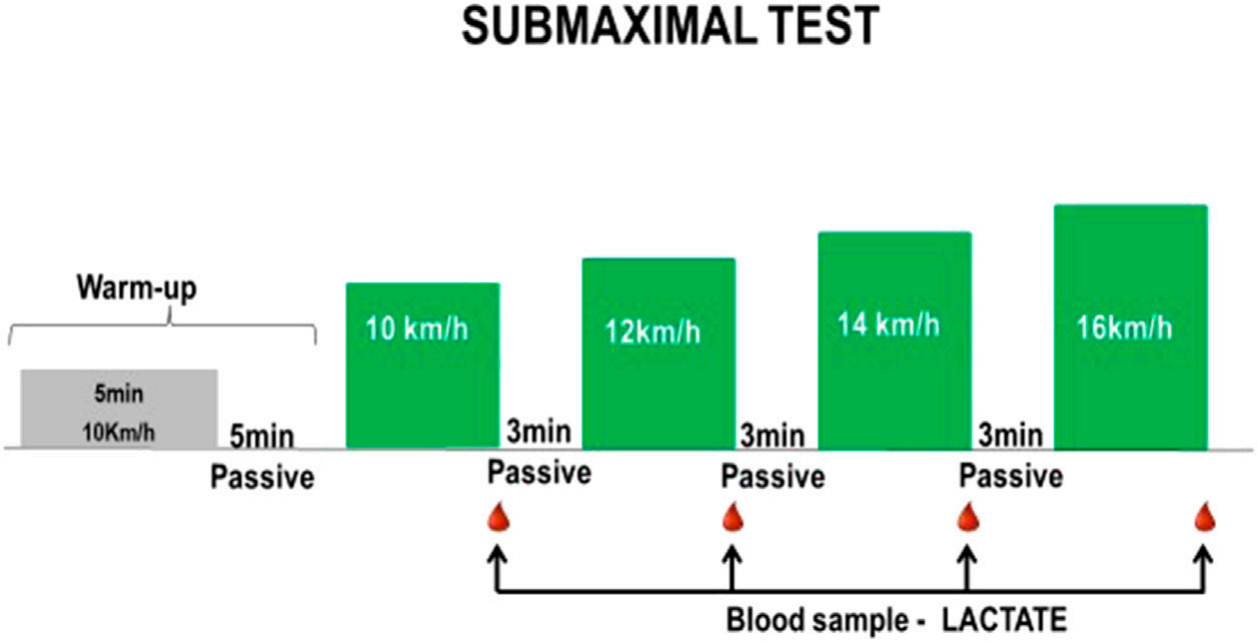
Model of submaximal test applied in mixed martial arts athletes in the −61 kg and −66 kg weight categories.

The submaximal tests consisted of 6-min stages. Each step has a constant speed. The initial speed was 10 km/h. The speed increases by 2 km/h in subsequent levels. The choice of the initial speed of the test, as well as the increments between each level, is based on the analysis of previous tests carried out by the subjects. There was a passive rest period between each plateau lasting 3 min. Before the first stage, the subjects performed a slight warm-up consisting of 3 min of running at the speed with which the test started, followed by 5 min of passive rest (Figure 1). Throughout the race, heart rate was measured during all the tests. After each effort level, blood lactate was measured. The test ended only when the subjects showed that it was impossible to continue the effort. The maximum speed each subject could sustain during the 6 min was considered the Maximum Aerobic Speed (VMA).

#### Supramaximal test

The supramaximal test was conducted until the participant reached exhaustion. The sole criterion for terminating the test was the apparent fatigue of the subject, indicated by their inability to sustain the effort at the chosen speed. An intensity level of 120% of the VMA (Velocity at Maximal Aerobic Speed) was selected to ensure that the supramaximal test lasted between 2 and 3 min, based on a review of the literature (Reis et al., 2005, Reis et al., 2007, Reis et al., 2013). VO2 (oxygen consumption) was continuously measured using a K4 gas analyzer from Cosmed, Italy. VO2 data were recorded for each respiratory cycle and then smoothed using a 10-s filter. At the beginning of the test, the treadmill was rapidly accelerated to reach the desired speed in approximately 5 s.

**FIGURE 2:**
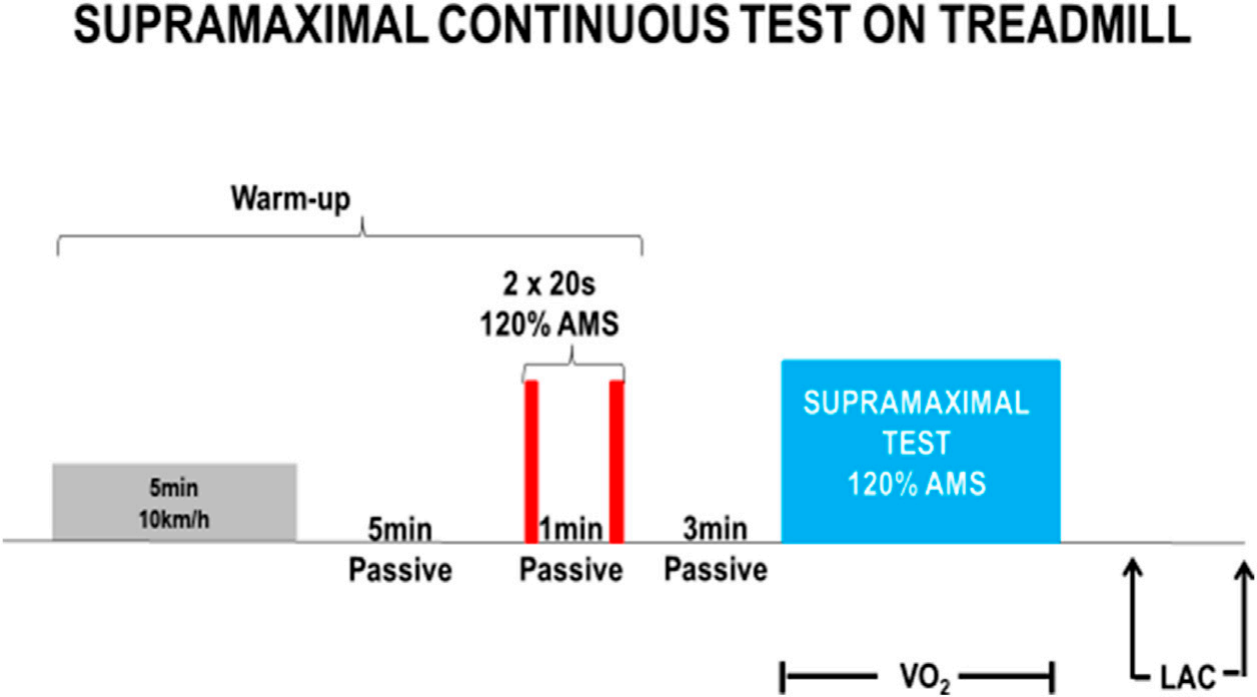
Model of subramaximal continuous test applied in mixed martial arts athletes in the −61 kg and −66 kg weight categories.

The participants’ warm-up consisted of 5 min of running at around 60% of their VMA, followed by a 5-min period of passive recovery. This was followed by two sets of 20-s runs at the test speed, with a 1-min recovery period between each set (see Figure 2). The test commenced 3 minutes after the warm-up. After completing the test, capillary blood samples were collected every 2 min to estimate the maximum blood lactate concentration.

#### Measures

All subjects underwent two exercise tests on a horizontal ATL KT10200 treadmill (Inbramed, Brazil). Blood collection was performed by a trained evaluator who had received specialized training and preparation. The study analyzed the progressive accumulation of lactate during submaximal tests by using linear interpolation to estimate the running speed corresponding to a lactate concentration of 4 mmol/L (V4). After each level of effort, blood lactate was measured using a Lactate Pro analyzer (Arkay, Brazil) that had been previously calibrated. Blood samples were obtained from the earlobe by an experienced technician.

The aerobic energy (EAER) in the supramaximal test was determined by integrating the measured VO2 during the test over the duration of the test. The anaerobic energy (EAN) in the supramaximal test was calculated based on the accumulated O2 deficit (DOA). The DOA included both the lactic and alactic components. Calculating the lactate component involved measuring the maximum post-exercise blood lactate concentration and converting it to energy, assuming an equivalent of 3 mL of O2 per mM of accumulated lactate (peak lactate concentration minus resting value). The alactic component was estimated based on the parameters defined by Di Prampero and Ferreti (1999). In more recent studies involving non-specialist active individuals (Zagatto et al., 2016; Redkva et al., 2018), a fixed value of 24 mL/kg-1 was used.The sum of the aerobic and anaerobic energy fractions represented the energy cost of the race.

### Statistical analysis

Descriptive data and comparisons between groups were made using the Statistical Package for Social Sciences 20.0 (SPSS). The Shapiro-Wilk test was also used to analyze the normality of the data. Our study performed the descriptive statistics, means, standard deviations (SD), and minimum and maximum values of the parametric variables. Pearson’s correlation was used to verify the correlation between the measurements obtained. Thus, the results are classified into 1) values <0, there is no agreement; 2) values between 0 and 0.20—weak agreement; 3) values between 0.21 and 0.40—distant agreement; 4) values between 0.41 and 0.60—moderate agreement; 5) values of 0.61–0.80—strong agreement; and 6) 0.81–1—almost perfect agreement. The significance level was *p* < 0.05.

## Results

When comparing the study variables by dividing the sample into two groups of different weight categories, no significant differences were detected between the variables (*p* ≤ 0.005), Table 1.

**TABLE 1:** Descriptive statistics of the maximal and submaximal tests in MMA athletes.

Variable	Group	Mean	SD	t-value	*p*-value
AMS	−61 kg	16.0	1.1		
	−66 kg	15.3	1.4	1.103	0.288
HRWP	−61 kg	139.4	12.5		
	−66 kg	139.6	22.6	−0.02	0.984
HRMAX	−61 kg	184.3	12.2		
	−66 kg	183.9	12.1	0.061	0.952
LAC1	−61 kg	10.6	4.7		
	−66 kg	9.1	2.3	0.798	0.443
LAC2	−61 kg	8.4	2.8		
	−66 kg	10.2	5.0	−0.93	0.37
LAC3	−61 kg	6.6	1.8		
	−66 kg	9.2	4.8	−1.18	0.287
LACmax	−61 kg	11.9	3.3		
	−66 kg	12.6	3.2	−0.46	0.656
Time	−61 kg	107.4	41.3		
	−66 kg	138.1	36.6	−1.62	0.128
VO_2_peak	−61 kg	43.5	3.5		
	−66 kg	40.7	4.1	1.49	0.157
VO_2_medium	−61 kg	35.6	3.6		
	−66 kg	34.0	3.4	0.903	0.381
LAC	−61 kg	18.3	6.9		
	−66 kg	14.3	3.8	1.458	0.174
DOA	−61 kg	42.3	6.9		
	−66 kg	38.6	3.5	1.4	0.191
AEST	−61 kg	64.6	26.6		
	−66 kg	78.9	25.4	−1.14	0.275
OEC	−61 kg	106.9	22.3		
	−66 kg	117.5	24.1	−0.94	0.362
%AEST	−61 kg	58.2	13.8		
	−66 kg	65.9	8.1	−1.38	0.196

Note: AMS, aerobic maximum speed; DOA, Deficit of O_2_ accumulated; OEC, overall energy cost; AEST, aerobic energy supramaximal test; LAC, lactate; HR, heart rate**;** HRWP, heart rate warm-up; VO_2_ = volume oxygen.

Table 2 presents the correlations between variables.

**TABLE 2:** Correlations between the dependent variables in MMA athletes.

Variables	AMS	Time	HRWP	HRMAX	LAC1	LAC2	LAC3	LACmax	VO^2^peak	VO_2_medium	LACT	DOA	AEST	OEC
HRWP	−0.200	0.150												
HRMAX	0.177	0.205	0.121											
LAC1	−0.178	0.016	−0.183	−0.039										
LAC2	0.189	0.029	0.377	0.463	−0.150									
LAC3	−0.584	0.612	0.373	0.284	−0.002	0.514								
LACmax	−0.466	0.473	0.111	0.270	.539^*^	.486^*^	.779^*^							
Time	−.848^**^	1.000^**^	0.150	0.205	0.016	0.029	0.612	0.473						
VO_2_peak	0.225	−0.012	−0.070	0.107	−0.342	0.070	−0.412	−0.162						
VO_2_medium	0.020	0.241	0.076	0.189	−0.330	0.152	−0.257	−0.029	.937^**^					
LACT	0.456	−.651^**^	−0.063	−0.070	0.462	0.264	−0.030	0.313	−0.118	−0.292				
DOA	0.438	−.628^**^	−0.040	−0.076	0.458	0.263	0.011	0.317	−0.109	−0.276	.997^**^			
AEST	−.772^**^	.967^**^	0.172	0.225	−0.081	0.078	0.498	0.423	0.224	0.467	−.652^**^	−.623^**^		
OEC	−.768^**^	.944^**^	0.185	0.237	0.018	0.151	0.548	.555^*^	0.227	0.463	−.499^*^	−0.466	.982^**^	
%AEST	−.686^**^	.909^**^	0.127	0.182	−0.209	−0.031	0.384	0.200	0.205	0.461	−.841^**^	−.831^**^	.926^**^	.849^**^

Note: AMS, aerobic maximum speed; DOA, Deficit of O_2_ accumulated; OEC, overall energy cost; AEST, aerobic energy supramaximal test; LAC, lactate; HR, heart rate**;** HRWP, heart rate warm-up; VO_2_ = volume oxygen.

The maximum heart rate was not related to any other variable. Meanwhile, the VO2 peak was negatively correlated with the professional experience number of MMA fights (r = −0.65, *p* < 0.01). The number of fights was also negatively correlated with anaerobic energy (r = −0.53, *p* < 0.05) and with the total energy cost (r = −0.54, *p* < 0.05).

## Discussion

The findings of our study revealed no significant differences in aerobic or anaerobic fitness when comparing two groups of MMA athletes from different weight categories. These results support the importance of incorporating submaximal and supramaximal tests in evaluating the physiological capabilities and performance potential of MMA athletes (Tota et al., 2019). Additionally, comparing weight divisions with close weights promotes fair contextual training, minimizes physical mismatches, and ensures athlete safety (Miarka et al., 2015). By implementing these practices, we can optimize training programs, enhance performance, and uphold the integrity of the sport of Mixed Martial Arts (Amtmann, 2003; Magnani Branco and Franchini, 2021).

Our study found no significant effects between the weight categories (−61 kg vs. −66 kg). However, there were several notable correlations between aerobic and anaerobic indicators, although maximum heart rate did not show any significant relationship with other variables. Interestingly, athletes with more experience exhibited lower aerobic power indicators (VO2 peak). The number of fights also negatively correlated with anaerobic energy and total energy cost.

MMA is a sport with a complex training system, requiring intensive physical training due to its intermittent nature, with fights consisting of 3–5 rounds of 5 min each. High aerobic power and anaerobic capacity values are believed to enable athletes to maintain high intensity, delay lactate accumulation, and facilitate recovery between rounds or intermittent actions such as kicks, punches, knees, and ground-based techniques (Miarka et al., 2016; Miarka et al., 2017). Conditioning is crucial to combat success (Kirk et al., 2020), and a weak aerobic component can limit performance (Plush et al., 2021).

Maximal oxygen consumption (VO2 peak) is considered the gold standard for assessing cardiorespiratory fitness in the general population (Craighead et al., 2019). In our study, we measured the cardiorespiratory fitness of MMA athletes using a specific ergometer (treadmill) and direct methods with increasing load (Chaabene et al., 2014). The results indicated that the MMA athletes had a mean VO2 peak of 42.01 ± 3.9 mL/kg/min. These values were similar to those reported for MMA athletes from northern Brazil (44.22 ± 6.69 mL/kg/min) (Oliveira et al., 2015) but lower than those reported in other studies (Schick et al., 2010; Alm, 2013; Lovell et al., 2013; Tota et al., 2014) conducted with MMA athletes. It is worth noting that our athletes were evaluated during a non-competitive period, and most of them had a body mass above their official combat weight. Therefore, in a competitive period, their VO2 peak could be 10% or higher than the measured value (Spanias et al., 2019).

The results of our study are consistent with typical values reported in the literature for athletes from other combat sports. The average VO2 peak values in these sports varied but generally fell within a range similar to the results obtained in our study (Ravier et al., 2006; Franchini et al., 2007; Chiodo et al., 2011; Farzad et al., 2011; Passelergue and Lac, 2012; Bruzas et al., 2014; Davis et al., 2014). Furthermore, our study measured the maximum aerobic speed of MMA athletes, finding an average speed of 16 km/h. These findings align closely with previous studies involving MMA athletes (Tota et al., 2014; Oliveira et al., 2016; Tota et al., 2019) as well as other combat sports such as wrestling, judo, jiu-jitsu, and kickboxing (Ravier et al., 2006; Franchini et al., 2007; Chiodo et al., 2011; Farzad et al., 2011; Passelergue and Lac, 2012; Bruzas et al., 2014; Davis et al., 2014).

The present study has methodological limitations that need to be considered; Sample size might make it difficult for researchers to extrapolate the findings to a larger group of MMA competitors. Additionally, the study does not include female athletes or athletes from other weight categories, which could provide a better understanding of the variables studied. Our study highlights significant differences in aerobic or anaerobic fitness when comparing MMA athletes from different weight categories. Incorporating submaximal and supramaximal tests in athlete evaluations provides a comprehensive understanding of their physiological capabilities and performance potential. Furthermore, comparing weight divisions with close weights promotes fair contextual training and ensures athlete safety. By implementing these practices, training programs can be optimized, performance can be enhanced, and the integrity of Mixed Martial Arts can be maintained.

It is important to note that the athletes included in this study were evaluated during a specific period not part of MMA’s competitive season. This distinction is essential as an athlete’s physical condition and performance may vary during different training phases and competitive periods. Therefore, the results obtained in this study may reflect the specific context in which the athletes were assessed, highlighting the significance of considering the competitive timeline in interpreting the findings.

## Conclusion

The findings of the present study suggest that there are no discernible differences in aerobic or anaerobic fitness when comparing two distinct groups of MMA athletes belonging to different weight categories, specifically the −61 kg and −66 kg divisions. Furthermore, noteworthy associations were observed between aerobic and anaerobic parameters, whereas no significant relationship was identified between maximum heart rate and other measured variables. And particular interest was the discovery that athletes with greater experience, as indicated by a higher number of fights, exhibited lower levels of aerobic capacity, as represented by peak VO2. Additionally, the number of fights displayed negative correlations with both anaerobic energy and total energy expenditure.

These findings may lend support to a training approach that incorporates athletes from both weight divisions in joint training sessions. However, it is essential to complement this unified training approach with weight-class-specific training programs custom-built to address the unique physical demands and requisites of each weight division. Making a balance between unified training methods and weight-specific conditioning can enable athletes to optimize their overall development and enhance their performance in the sport of MMA.

The study’s practical applications embrace a broad range of benefits for athletes, coaches, and sports scientists. These applications include optimizing training programs made to athletes’ specific physiological characteristics, aiding athletes in choosing the most suitable weight category for competition, improving performance by addressing areas of weakness, implementing injury prevention strategies, identifying talent for recruitment, making it easy to monitoring performance, promoting fair competition based on weight categories to reduce injury risks, and advancing the field of sports science with valuable insights into MMA athletes’ physiological profiles. These applications collectively enhance athlete development and the overall understanding of combat sports physiology.

## Data Availability

The original contributions presented in the study are included in the article/Supplementary material, further inquiries can be directed to the corresponding author.
