# Corrigendum: Enhancing performance: unveiling the physiological impact of submaximal and supramaximal tests on mixed martial arts athletes in the −61 kg and −66 kg weight divisions

**DOI:** 10.3389/fphys.2024.1447631

**Published:** 2024-07-11

**Authors:** Aleksandro Ferreria Gonçalves, Bianca Miarka, Clóvis de Albuquerque Maurício, Rafael Pereira Azevedo Teixeira, Ciro José Brito, Diego Ignácio Valenzuela Pérez, Maamer Slimani, Hela Znazen, Nicola Luigi Bragazzi, Victor Machado Reis

**Affiliations:** ^1^ Research Center in Sports Sciences, Health Sciences and Human Development, University of Trás-os-Montes and Alto Douro, Vila Real, Portugal; ^2^ Laboratory of Psychophysiology and Performance in Sports and Combats, Graduate Program in Physical Education, Federal University of Rio de Janeiro, Rio de Janeiro, Brazil; ^3^ Sciences of Physical Activity, Sports and Health School, Faculty of Medical Sciences, Universidad de Santiago de Chile, Santiago, Chile; ^4^ Escuela de Kinesiología, Facultad de Salud, Universidad Santo Tomás, Santiago, Chile; ^5^ School of Public Health, Department of Health Sciences (DISSAL), Genoa University, Genova, Italy; ^6^ Department of Physical Education and Sport, College of Education, Taif University, Taif, Saudi Arabia; ^7^ Laboratory for Industrial and Applied Mathematics (LIAM), Department of Mathematics and Statistics, York University, Toronto, ON, Canada

**Keywords:** aerobic system, anaerobic system, martial arts, oxidative system, glycolysis

In the published article, there was an error in the **Funding** statement. The statement “VMR was funded by FCT—Fundação para a Ciência e Tecnologia (UID04045/2020)” was incomplete. The correct **Funding** statement appears below.

